# The impact of moving to a 12h shift pattern on employee wellbeing: A qualitative study in an acute mental health setting

**DOI:** 10.1016/j.ijnurstu.2020.103699

**Published:** 2020-12

**Authors:** Jane Suter, Tina Kowalski, Misael Anaya-Montes, Martin Chalkley, Rowena Jacobs, Idaira Rodriguez-Santana

**Affiliations:** a The York Management School, University of York, Freboys Lane, Heslington, York YO10 5GD, UK; b Centre for Health Economics, Centre for Health Economics, University of York, Heslington, York, YO10 5DD, UK

**Keywords:** Extended working hours, 12 h shifts, Wellbeing, Mental health services, Qualitative, Work-life balance, Social support

## Abstract

**Background:**

Against a backdrop of increasing demand for mental health services, and difficulties in recruitment and retention of mental health staff, employers may consider implementation of 12 h shifts to reduce wage costs. Mixed evidence regarding the impact of 12 h shifts may arise because research is conducted in divergent contexts. Much existing research is cross sectional in design and evaluates impact during the honeymoon phase of implementation. Previous research has not examined the impact of 12 h shifts in mental health service settings.

**Objective:**

To evaluate how employees in acute mental health settings adapt and respond to a new 12 h shift system from a wellbeing perspective.

**Design:**

A qualitative approach was adopted to enable analysis of subjective employee experiences of changes to organisation contextual features arising from the shift pattern change, and to explore how this shapes wellbeing.

**Setting(s):**

Six acute mental health wards in the same geographical area of a large mental health care provider within the National Health Service in England.

**Participants:**

70 participants including modern matrons, ward managers, clinical leads, staff nurses and healthcare assistants.

**Methods:**

Semi-structured interviews with 35 participants at 6 months post-implementation of a new 12 h shift pattern, with a further 35 interviewed at 12 months post-implementation.

**Results:**

Thematic analysis identified unintended consequences of 12 h shifts as these patterns changed roles and the delivery of care, diminishing perceptions of quality of patient care, opportunities for social support, with reports of pacing work to preserve emotional and physical stamina. These features were moderated by older age, commitment to the public healthcare sector, and fit to individual circumstances in the non-work domain leading to divergent work-life balance outcomes.

**Conclusions:**

Findings indicate potential exists for differential wellbeing outcomes of a 12 h shift pattern and negative effects are exacerbated in a stressful and dynamic acute mental health ward context. In a tight labour market with an ageing workforce, employee flexibility and choice are key to retention and wellbeing. Compulsory 12 h shift patterns should be avoided in this setting.

**What is already known about the topic?**

• Evidence for the impact of 12 h shifts on organisational and employee outcomes is mixed and the complexity of the issue is poorly understood.
• 12 h shifts can increase sickness absence in general nursing indicating these extended shift patterns can affect wellbeing.

**What this paper adds**

• Findings demonstrate how a 12 h shift pattern can reduce perceived quality of patient care and opportunities for non-routine care, increase anxiety if staff feel out of touch with clinical knowledge following extended time away from a dynamic ward environment, and encourage pacing of work.
• Illustrates how the removal of a longer middle handover can alter resources such as social support that could mitigate strain arising from high job demands, and diminish opportunities to reflect upon professional practice.
• Identifies potential modifiers to employee outcomes of a 12 h shift pattern.

## 1 Introduction

Increasing demand for mental health services in the UK, estimated to increase by 2 million service users by 2030 (Mental Health Foundation, 2013), alongside difficulties in recruitment and retention of mental health staff, have led to severe financial pressures for mental health service providers (UNISON, 2017). In contrast to adult general nursing, the mental health and learning disabilities workforce in England has decreased over the past decade, with an estimated 11% of nursing posts vacant and a negative net effect of staff turnover (−4% annually as compared to +2% for Adult Nursing) (Health Education England, 2017). With an emphasis on community care for mental health service users, experienced staff are leaving acute inpatient settings with evidence suggesting 25% of inpatient staff moved to newly created community teams (Sainsbury Centre for Mental Health, 2005). Additionally, there is concern that the removal of nursing bursaries may disproportionately affect mental health nursing numbers (Royal College of Nursing, 2018a). This shifting demand for mental health services and tight labour market are worsened by a workplace context characterised by strain. Whilst mental health nurses share many of the same stressors as their counterparts in general nursing, they face additional stressors thought to arise from intense interactions with patients, many of whom are violent and disruptive (Edwards and Burnard, 2003), with staff burnout a particular problem (Morse, Salyers, Rollins, Monroe-DeVita, and Pfahler, 2012). Evidence suggests mental health nurses suffer a high risk of physical assault (Renwick et al., 2016), and have to contend with the use of coercive measures such as restraint and detention of patients (Bonner, Lowe, Rawcliffe, and Wellman, 2002), and the need for continuous monitoring of patients at risk of self-harm and suicide (Hagen, Knizek, and Hjelmeland, 2017). The use of emotional labour is inherent in mental health roles as staff have to perform positive or neutral emotions when caring for patients who are distressed or aggressive, with research highlighting the association of prolonged performance of emotional labour with stress (Mann and Cowburn, 2005) and alienation from the profession (Moloney, Boxall, Parsons, and Cheung, 2018).

Nurses working 12 h shifts is a growing trend in the UK with an estimated 31% working these shifts in 2005, rising to 52% in 2009 (Ball, Dall'Ora, and Griffiths, 2015). Data from 12 European countries indicates 14% of acute nurses were working 12 or more hours per shift (Griffiths et al., 2014). With universal pressures on health services, this is a model employers may increasingly consider to reduce wage costs, as organisations operating around the clock are serviced by two shifts instead of three, and removes a longer middle handover where more staff are present. Evidence on the impact of extending working hours (e.g. increase from eight to 12 h shifts) is mixed, and focuses on potential negative consequences e.g. increased accidents, fatigue, adverse effects on health and wellbeing, and absenteeism (Dall'Ora, Ball, Recio-Saucedo, and Griffiths, 2016). Research highlights potential benefits to employers (fewer handovers, less overtime) and to workers (less travel time and longer periods between shift patterns (Dwyer, Jamieson, Moxham, Austen, and Smith, 2007; Knauth, 2007; Richardson, Turnock, Harris, Finley, and Carson, 2007), and perceptions of better organisation and delivery of care (Baillie and Thomas, 2019). However, a 2015 review concludes insufficient evidence for either benefits, nor a detrimental impact, of 12 h shifts and the complexity of the issue is poorly understood (Harris, Sims, Parr, and Davies, 2015). In part, this could be due to the variety of sample populations and settings where research has been conducted. Recent research examining the impact of 12-hour shifts in general nursing have indicated a negative impact on sickness absence (Dall'Ora et al., 2019a). Dall'Ora, Griffiths, Emmanuel, Rafferty, and Ewings (2019b) question the assumption that a longer handover period characteristic of an 8 h shift pattern is unproductive, and report cross-sectional survey data from 12 European countries that show nurses working 12 h shifts were less likely to report opportunities for training and discussion of patient care compared to nurses working 8 h shifts. Extant research emphasises and quantifies distinct outcomes for individuals or problems for the employing organisation in terms of impact on performance, but less is known about what moderates these outcomes or the nuances of how individuals respond to 12 h shifts from a wellbeing perspective, a limitation that can be overcome with qualitative research.

The present article qualitatively examines how staff working on acute wards in a large mental health organisation in England respond to the introduction of 12 h shifts and the way in which this affects employee wellbeing. See Rodriguez Santana et al. (2020) for a quantitative analysis of the impact of 12 h shifts on sickness absence amongst the same population. Understanding how 12 h shifts are experienced in an inherently stressful context is informative as longer periods of exposure to this setting may lead to severe consequences for employee wellbeing. Extant research examining the impact of organisational change on employee wellbeing typically focusses on broad, objective measures with less attention paid to subjective experiences, potentially resulting in an underestimation of the effects of organisational change to individual wellbeing (Rafferty and Jimmieson, 2017). Following an evaluation of the impact of organisational change on employee wellbeing in healthcare organisations, Gibbs, Loretto, Kowalski, and Platt (2011) call for qualitative evaluations to compliment quantitative research that highlights the nature and extent of stressors associated with organisational change. As such, this study adds to evidence through reporting rich data to illuminate the experiences and perceptions of employees moving to a new 12 h shift pattern and aids understanding of objective outcomes.

A review of extended work patterns by Tucker (2006) calls for evaluations of changes to work schedules beyond the first 6 months post-implementation to allow a new shift regime to embed into practice. Tucker (2006) refers to the ‘honeymoon period’ of change soon after extended shifts are introduced where employees experience ‘euphoria’ in response to additional days off and argues any impact on wellbeing can take time to manifest. The present study aims to address Tucker's observation by moving beyond cross-sectional analysis, addressing limitations of extant qualitative research with a single data collection point (Baillie and Thomas, 2019; Thompson, Schneider, and Duke, 2017), by evaluating the impact of 12 h shifts on employee wellbeing at six months post-implementation, with a follow-up at one year.

This is the first study to evaluate the impact of 12 h shifts in an acute mental health service setting, which we argue has additional stressors to general nursing and as such is a more intense environment for 12 h shift patterns, particularly over time. A qualitative approach extends understanding by illustrating how the impact of extended shifts on wellbeing may go beyond any fatigue associated with longer work hours, as the demands of a 12 h shift pattern are shaped by features specific to the organisational and workforce context. We identify aspects of the workplace context that change with a move to 12 h shifts, illustrating how a 12 h shift pattern may be detrimental to perceptions of the quality of patient care, social support, and the ability to maintain energy and focus for extended periods. In our sample, these features were further moderated by older age, a commitment to the public healthcare sector, and fit to individual circumstances in the non-work domain leading to divergent work-life balance outcomes. Together these findings imply potential exists for differential wellbeing outcomes for employees and organisational contexts.

## 2 Methods

A qualitative methodology was employed to explore employee subjective experiences of organisational change, in this case, a mandatory move to 12 h shift patterns to align with existing schedules of a new management Trust. This paper follows the consolidated criteria for reporting qualitative studies (COREQ) (Tong, Sainsbury, and Craig, 2007). Data were drawn from six mental health wards in the same geographical area of a large mental health care provider within the National Health Service (NHS) in England. The sample included staff from three Adult Mental Health wards, two Older People Services wards, and one Learning Disability Services ward. In-depth interviews explored how participants perceive the impact of the shift change on health, wellbeing and organisational outcomes. A purposive sampling framework was used to collect data in two fieldwork phases from five layers of participants (modern matrons, ward managers, clinical leads, staff nurses and healthcare assistants). Thirty-five staff and managers were interviewed in phase 1 (6 months post-implementation) and a further 35 in phase 2 (1 year post-implementation). Both phases had representation from each of the wards. Phase 1 interviews were conducted September to October 2018. Phase 2 interviews were held February to March 2019. The age of participants ranged from 25 to 69 years with an average age of 45. Interviews were held in a private office in the workplace. Ward managers advised staff the research was taking place and facilitated cover to allow interviews during their shift, with participants self-selecting (Saunders, 2012). For practical reasons, and to ensure each phase had a sample size that reached data saturation, Phase 1 and 2 samples were different. Two phases of interviews enabled comparison of themes from each cohort, strengthening the robustness of findings. Table 1 provides sample characteristics.

**Table 1:** Phase 1 and 2 interview sample characteristics.

	Phase 1 (P1)	Phase 2 (P2)
Gender:		
Male	10	6
Female	25	29
Job role:		
Health care assistant	11	19
Nurse	15	10
Ward manager	6	6
Other senior managers	3	0
Age range:		
18–29	7	5
30–39	2	6
40–49	4	8
50–59	9	10
60+	4	0
n/a (managers)	9	6
Length of service in NHS:		
Less than 5 years	7	9
6–14 years	6	13
More than 15 years	22	13

**Note:** In the Results section, the Participant ID consists of the job role, interview number and phase number, e.g. (Nurse 5, P1).

This study received ethics approval from the Health Research Authority. As part of the informed consent process, participants were given a project information sheet to outline the purpose of the project and reassure participants of confidentiality and anonymity. Data were only accessible to the research team, and participants were advised at interview that potential identifiers would be removed from outputs. Duration of interviews varied between 23 and 95 min, and covered a range of topics, beginning with general questions about the nature of the job and broader context around organisational change in the NHS, before focussing on staff experience of working 12 h shifts and implications this had for them (for example, in terms of workload, working environment and relationships with others). A topic guide was used, with scope for participants to detract from this with other issues pertinent to them. Interviews were audio recorded with the exception of three participants who did not consent to this. A final study report was sent to all participating ward managers, the regional head of nursing, and presented to the Trust Board.

### 2.1 Research team and reflexivity

The interviewers (first and second author) comprised two female academics educated to PhD level with expertise in employment research, with no prior relationship with the case study organisation, little prior knowledge of the mental healthcare context and no experience of the working conditions. This outsider perspective is arguably a strength of the study. Cassell (2015) cites reflexivity as a fundamental component of interview research and acknowledges the importance of ‘identity work’ to help interpret interview data as an ongoing process, both in terms of the narrative of the interview itself and subsequent analysis. In this vein, it is important to note the research team was independent from the NHS and did not meet with participants before the interview. This may have influenced what participants shared and how they framed their responses, something the interviewers reflected upon during the analysis phase.

### 2.2 Data analysis

Data saturation is an indicator of qualitative rigour (Morse, 2015) with meaningful saturation thought to be between 16 and 25 interviews (Hennink, Kaiser, and Marconi, 2017). Data saturation was agreed by both interviewers at around 20 interviews, facilitated by concurrent data collection and analysis. We further increased the sample size to ensure proportionate representation of the six wards and different job levels.

Data were managed using Nvivo software (QSR International, Cambridge, MA). Analysis occurred at a number of levels, using a constant comparative process undertaken by repeated listening to recorded interviews and reading of transcripts, coding these iteratively and thematically. First level coding included consequences of the shift change, positive and negative, coping mechanisms, and wellbeing and organisational outcomes. Informed by this first level coding, a conceptual framework was developed (Silverman, 2016) and refined with ongoing analysis and coding to identify emerging themes derived from the data. Discussions between the two interviewers facilitated the process in terms of an aide memoire and providing clarification on the interpretation of data. This reflexivity served to minimise potential bias in the analysis of interview data (CASP, 2018).

Analysis of data is categorised under two overarching concepts: unintended consequences of adopting a 12 h shift pattern, and factors influencing responses to a 12 h shift pattern. Within these, six dominant themes emerged which we labelled ‘perceptions of quality of patient care’, ‘stamina and pacing of work’ and ‘social support’ (relating to unintended consequences), and ‘older age’, ‘public healthcare commitment’ and ‘work-life balance’ (relating to factors influencing responses). Below we present an overview of the study context before exploring these key themes.

## 3 Results

### 3.1 Context - The move to 12h shift patterns

#### 3.1.1 Consultation process

Following a management change from one NHS Trust to another, a consultation on the introduction of 12 h shift patterns was undertaken. Staff received letters detailing the proposed change, and met with senior management. Despite mixed support following a vote on staff preferences, a 12 h shift pattern was implemented. Few participants recalled feedback on the outcome to this process nor a formal procedure to give feedback on the change; instead, many gave informal feedback to colleagues and ward managers. Generally, participants felt they had little influence over Trust wide policy on working time policy. Lead in time for the shift pattern change varied across wards, with some wards moving relatively quickly after it was confirmed, with the last ward 6 months after the consultation took place.

Irrespective of how respondents felt about the shifts themselves, many viewed the change as imposed and perceived it as a cost saving exercise, leaving some staff frustrated:

*“The only reason they put us on 12 h shifts is to save money really, 'cause, you know, you don't get the overlap of staff. It wasn't to benefit us; it would be to benefit them” Healthcare assistant 2_P1*

*“They did a consultation with staff and they asked them to vote and the vote didn't go the way the Trust wanted it to go, they expected that staff would ask to do long days and they didn't…so, yeah, you could say they consulted, but I don't think it was the best of consultations” Manager 3_P1*

#### 3.1.2 The new shift pattern

Prior to the new shift pattern, the majority of staff worked a short shift pattern (5 × 8 h shifts over 7 days). There was also flexibility for staff to request a mixture of shift lengths, for example, some longer shifts, mostly early or late shifts, or majority night shifts, at the discretion of ward managers. The new pattern required full-time staff to work 3 × 12 h shifts over 7 days and an additional 12 h shift per month to make up the same working hours.

Under the old working pattern, there were handovers at the start of the early, late and night shifts. With the move to 12 h shift working, the middle handover of up to 2 h was lost. Excess staff in an extended crossover period facilitated team meetings, clinical supervision, training, and patient activities such as escorting patients outside, to appointments or to help patients with ‘non-essential’ care such as painting nails or going to the hairdressers, something patients valued and which gave staff job satisfaction.

*“[sighs] it's just, it just impacts on everything, patient care, what about the way in which work is organised? So things like supervisions, training opportunities, take patients out…the staff, some staff get a bit short tempered because the long days, it's just, it's hard” (Healthcare Assistant 30_P2).*

The loss of this middle handover meant opportunities for staff involvement and development were lost, or severely disrupted, with potential implications for quality of patient care and feelings of job satisfaction for staff.

### 3.2 Unintended consequences of adopting a 12h shift pattern

#### 3.2.1 Perceptions of quality of patient care

Continuity of care reportedly changed in both positive and negative ways. Some participants reflected on how continuity of care improves with a 12 h shift pattern, as fewer shift changes are less disruptive for both patients and staff. As one nurse revealed:

*“I'm all settled doing longer shifts because you're just more settled into what you need to do. You're able to finish the jobs you've got; that's a big plus as people were always staying behind with shorter shifts” (Nurse 5_P1)*

Others emphasised negative affects to continuity of care over longer periods, as patients may not see familiar staff or a named nurse, particularly as turnaround for acute wards can be quick. At follow up interviews, one manager reflected on how the compressed workweek associated with a 12 h shift pattern altered the availability of experienced nurses present on the ward throughout the week:

*“I miss having my staff nurses around five days a week to have more of a grasp on what's happening with the client base, because turnover's pretty quick, we get people in and out very quickly. If you have a couple of long days and then have three or four days off, the client group can change quite dramatically, and it feels to me that sometimes people are just playing catch-up all the time” (Manager 10_P2*)

This excerpt also exposes how productivity might be impeded following extended time away from the workplace, as staff spend time familiarising or reacquainting themselves with care plans and critical incidents. Relatedly, both staff and managers reflected on how feeling out of touch with clinical knowledge, due to the dynamic acuity of the ward environment, could increase anxiety. Participants with additional responsibilities, such as to lead dissemination of information to other staff or for dispensing medicines, emphasised this:

*“I was worried about making errors. I'd made a drug error and so all those, sort of, anxieties were kicking in. Anxious. Yeah, particularly on a Monday, because everybody's here on a Monday and, if you're in charge of the report out and lots has happened, and people are asking questions, and you haven't got the answers…so yeah, I started getting more anxiety” (Nurse 21_P2)*

Diminished time to deliver ‘non-essential’ care such as accompanying patients to the shops, or personal care such as attending to a patient's hair and nails, was reported by some staff because the removal of the middle handover period limited opportunities for this type of care. Staff and managers revealed how this reduced job satisfaction and affected wellbeing. As one nurse described:

*“I've had several staff in tears, and come in the next day and said, 'I was crying last night when I got home', because there's a lot of older staff here…they're used to being able to do the things that we want to do: give people a bath, a shower, make sure they've had a shave, things like that; now we just don't have the time to do that, and they're very upset that they're leaving people unkempt” (Nurse 2_P1)*

#### 3.2.2 Stamina and pacing of work

A recurring theme was a need for physical and emotional stamina when working on acute mental health wards. The patient mix and acuity was perceived as important for the ability to tolerate 12 h shifts, as an extended shift with noisy or aggressive patients was particularly stressful. By Phase 2, rather than adapting, some staff remained exhausted by 12 h shifts:

*“We've got four on within eyesight…these gentlemen, walk around constantly. So there's staff who are just with them all day, going from obs [eyesight observations] to obs to obs, with the odd gap, walking around all day. So people are exhausted by the end of the day” (Nurse1_P2)*

A 12 h shift pattern therefore had implications for how staff organised their work and went about tasks with participants emphasising how they would pace their work to preserve physical and mental energy. As one nurse explained:

*“The difference is quite simple. If I'm coming in at half seven and going home at three, I think, right, do you know what, eight hours, I'll give it my all, I'm not back till tomorrow dinner time, or tomorrow morning. But now I look at eight o'clock at night and think oh, my God, how am I going to get there…it's a long way ahead of you…you can only keep going at a certain rate for so long, and then you start to, - and I'm not used to saying that to be honest with you; I didn't think I'd be sat here saying that. I can usually get through it and keep a smile on my face, but it's difficult” (Nurse 2_P1)*

This excerpt also exposes the potential stress of a prolonged performance of emotional labour and how ‘keeping a smile on one's face’ over an extended shift becomes difficult. Relatedly, participants revealed how the nature of tasks they felt able to complete varied over the course of a 12 h shift:

*“when you're here for 12 h you have a set number of tasks you want to do during the day and it does get to the point in the afternoon where you still have jobs to do but it's almost like your brain turns to mush and you can only do the most basic things” (Nurse 4_P1)*

Participants further revealed how the intensity of work over a 12 h shift depletes internal resources. The excerpt below highlights how staff struggle to maintain stamina for 12 h and how this affects patient care:

*“I start whizzing around and that's not a good time for me because if I whizz too much then that's when I will break. And I will need to have some time off and I can't keep doing that…I just think, oh, I'm going have to take a step back and think, I can't do everything. But then these people that I'm looking after, they're not getting 100 per cent because we're knackered” (Healthcare Assistant 3_P1)*

Below, a nurse describes the intensity of work over a 12 h shift and how tolerance wears down, particularly when working with agency staff, a common feature of working on acute wards:

*“A 12-hour shift with certain staff can be really stressful. Last week, I had staff that weren't regular on the ward and I was just inundated every five minutes with information that I didn't really need to be told, but they felt they had to tell me. So you're input goes up and up, and you're just like a blotting paper absorbing all this useless information that you don't need. But you're taking it on board till you get quite worn down with it, so looking at 12-hour shifts that way, you do absorb a lot more” (Nurse 3_P1)*

#### 3.2.3 Social support and reflection

Participants identified social support as an important mechanism for reflecting on stressful instances, and in enhancing their ability to cope with job demands. Most participants perceive the 12 h shift system as leading to diminished access to social support in a variety of ways relating to the opportunity for, and nature of, support. Participants recounted reduced opportunities for informal social support through shared reflection on patient care and stressful incidences:

*“It's reducing time for staff to have that quality time with each other, that time to think…There's that time that has just been taken away. That's caused an additional stress to staff” (Manager 1_P1)*

Some participants expressed feelings of isolation, as they would often have extended periods between seeing colleagues due to the scheduling of 12 h shift patterns. This isolation was heightened by working alongside agency workers, and the increase in job demands that accompanied this. For some, the introduction of 12 h shifts had negatively affected relationships with colleagues. The example below highlights how workers who perceive themselves as more efficient, can have heightened negative attitudes toward colleagues they view as less productive and how this reduces cooperation and discretionary behaviours during an extended shift:

*“Rather than just accept that I'm coming in on a long day and I'll do all your work for you, just so we'll all be friends. Like, I can't do that, because it's a long day. A short shift I would have done that. Now I realise you were taking the mickey a little bit…So there's no teamwork anymore. There's no support” (Healthcare assistant 4_P1)*

In an attempt to adapt to these new circumstances, one ward manager allocated protected time at the end of shifts in a bid to formalise ‘reflective practice’ where staff reflected on situations arising on the shift and submitted a written record of this. However the loss of opportunities for support from informal social interaction are unlikely to be captured by a formal process or viewed as comparable by employees:

*“You will sit with someone and you'll, kind of, open it up. And I suppose really that's probably not even documented because you just do it as part of being two colleagues together sat in a computer room in the office doing your notes…what situation (formal) reflection has done is, try to cover what we've lost in the middle of the day, which I don't think it has because it's structured and it's something to follow” (Nurse 9_P1)*

In addition, more formal routes to support, for example at team meetings, were restricted because of difficulties in scheduling meetings as it was rare to have excess staff on the ward at any one time. Instead, managers relied on informal communication and limited meetings to a small number of staff on an ad hoc basis. Moreover brief handovers at the beginning and end of the day constrained potential social support as these handovers focussed on efficient communication of clinical information required for the next shift. Staff also reported reduced opportunities for sharing good practice with colleagues and less ‘downtime’ to reflect.

This section has depicted the way in which work demands changed and how workers responded, highlighting unintended consequences of the introduction of a 12 h shift system and how these can increase stress, diminishing wellbeing.

### 3.3 Significant factors influencing responses to a 12 h shift pattern

Individual characteristics (e.g. age), coupled with individual circumstances (e.g. caring responsibilities) affected how staff adapted and responded to the change in shift pattern. Those who responded positively and felt little adverse impact on their wellbeing reported various reasons for this, mainly relating to the pay-off of a compressed workweek. However, rather than ‘enjoying’ these shifts, many participants had come to accept a 12 h shift pattern and found ways to make it work for them, and expressed good levels of job satisfaction and positive work-life balance. Conversely, where participants spoke about adapting and responding more negatively, feelings of frustration and disengagement were revealed. In this section, three themes reported highlight significant features of the empirical context shaping how participants adapted to a 12 h shift pattern. These features are unlikely to be the only factors shaping employee wellbeing in relation to 12 h shifts, however our analysis identifies those most relevant to participants within this specific context. These are older age, public healthcare sector commitment, and work-life balance.

#### 3.3.1 Older age

A number of respondents referred to how their older age affected their ability to adapt to the new shift pattern. One older nurse highlighted:

*“If you're getting a bit older, I mean, I've noticed myself in the last couple of years, if you do a lot of days together your legs start aching and your back starts hurting” (Healthcare assistant 19_P2)*

Although coping with fatigue was not an issue restricted to older staff, as many younger staff commented on exhaustion, this was a theme strongly emphasised by older participants. Some participants were approaching retirement age and had been keen to join the relief pool of workers. Amongst these, many revealed a requirement to work a 12 h shift had persuaded them not to return. Some older workers emphasised how flexibility in shift patterns for retiring workers could facilitate staying in the role for longer, albeit on a part-time basis. Managers were conscious of the loss of expertise if older workers were not retained and subsequent impact on quality of patient care and mentoring of less experienced staff.

Data reveals the impact of 12 h shifts for older workers relates to both within and between shift stamina. Some perspectives, such as those above, focused predominantly on the ability to complete a shift comfortably, whilst others emphasised the impact on recovery time between shifts and how this lengthened as they got older. As an older nurse in her sixties recounted:

*“You've got an extra day at home, but for me it takes that extra day to recover, only because of my age. I'm a lot older than the other nurses, so I do take a while to recover from a 12* h *shift…and because of my age, my legs ache terrible at the end of the day, it's a long day to be on your feet, a long, long day, especially when you've had some back and leg problems, I struggle, I get to about six o'clock and I'm starting to drag myself around*” (Nurse 3_P1)

Physicality of work was an issue raised frequently and not only by older workers, with a number of participants commenting on how the physical component of the role had intensified in recent years as a consequence of the Transforming Care agenda which seeks to treat more patients in the community. Those admitted to acute wards tend to require more intensive care and with unpredictable and volatile behaviour restraining patients is a regular part of the job. Older participants revealed how this physicality inherent in roles on acute mental health wards is challenging as they get older and more so over a 12 h shift.

#### 3.3.2 Public healthcare commitment

Commitment to the public healthcare sector was a theme we used to categorise the range of perspectives relating to high levels of occupational and sector commitment expressed by participants, and which seemed to shape how staff adapted and responded to the shift pattern change. Many participants had worked in the NHS for most of their working lives (see Table 1) and felt a strong sense of attachment to it, as exemplified by a healthcare assistant participant:

*“I would have left a long time ago if it wasn't for the fact that I love the job. So, I feel like, with everything that the Trust and the NHS has been through, if you work in this sector, if you're still in it now, it's because you want to be, because you love the job. Because there's been too much that's happened for people - we've lost so many people that have been put under so much pressure. So, if they've made it through this far, I think it's because of a true love of your job” (Healthcare Assistant 4_P1)*

Another manifestation of this commitment was highlighted by a significant number of participants who reported they had to be ‘seen to be coping’. When asked how staff were adapting to 12 h shifts, a common response would be *“It's fine! …”.* If they thought their colleagues were coping, they also wanted to be seen as coping. As one manager revealed:

*“There's that tendency to think that that's their job, that's their role, they've got to be seen to be coping because actually if they're not seen to be coping, it doesn't have a good impact on the rest of the crew, and that's the angle I think most of them are coming from” (Manager 2_P1)*

This commitment remained evident at follow up interviews, with a sense of staff ‘just getting on with it’ prevalent:

*“I think they do accept that they just need to, yeah, knuckle down and get on with it 'cause it, it's kind of the nature of working for the NHS” (Healthcare assistant 17_P2)*

Indeed, there was little illusion amongst staff around the challenging nature of their work environment, or of the broader challenges faced by the NHS, yet we observed that many participants were reluctant to complain about their circumstances and were persisting with working 12 h shifts, even though they may find it physically and emotionally exhausting. A number of participants reported being physically attacked, or injured at work, yet for many they saw it as ‘just part of the role’. Comments emerged around partners or family members becoming angry when their loved ones were assaulted, with participants emphasising how those outside the profession ‘just don't understand’, again reiterating a commitment to the role, which may not be evident in other professions or contexts.

#### 3.3.3 Work-life balance

Some staff responded positively to the move to 12 h shifts as these patterns aided work-life balance. For others, focusing on the trade-off of additional days off helped mitigate any adverse effects. The following excerpt illustrates how respondents sought to re-evaluate negative perceptions of 12 h shifts by focusing on the compensatory benefits to work-life balance:

*“Cause you're doing three days here, you've got four days away, and I think - well, for myself I need it after our fellows here, especially if you've had a rough day…No, if I'm dead on my feet when I walk out of here, by the time I've got to my car I'm fine, [chuckling] because I know I'm going to be off” (Healthcare Assistant 2 _P1)*

However, despite expectations of satisfaction with a compressed work week the experiences of many respondents were negative as some revealed how being in the workplace for 12 h restricted out of work activities solely to non-workdays. With shorter shifts there is at least an opportunity to carry out personal tasks or to spend time with family before or after a shift. As one nurse explained:

*"They keep saying, 'Not at work again, Mummy. Will we see you tomorrow morning?', I'm like, 'No, I'm really sorry, I'll be gone to work by…', and by the time I get home, one of them is asleep in bed…I have noticed my youngest one is staying up to try and see me but then she's shattered the next day and it's not fair for school. So two long days together is even worse because then they don't see me for like two whole days, it's awful” (Healthcare assistant 29_P2)*

Restrictions to the lives outside of work were not limited to childcare, with participants reflecting on how longer shifts and a compressed workweek affected a range of personal activities such as opportunities to engage in hobbies, socialising or exercise. As a participant without childcare responsibilities commented:

*“I've found that my home-life, work-life balance is not as it used to be…we [partner] will not see each other at all for four days…I think the shorter shifts, at least with them you've actually got more time at home…I used to box a lot…boxing starts at 6:00 and you doesn't finish till 8:00, so I miss it. I used to like to go swimming on a morning before a late shift, but obviously we start at half seven in the morning, so I can't go swimming in the morning anymore, apart from on my days off. Quite often I'm knackered by then and I don't want to do owt” (Healthcare assistant 14_P2)*

This excerpt illustrates how despite supposed improvements to work-life balance on offer with a compressed workweek, a 12 h shift pattern can constrain non-work activities, indicating choice and fit to individual circumstances are essential for potential benefits.

## 4 Discussion

This research sought to examine the impact of extended shifts on wellbeing through employee experiences of change. At a time of increasing demands for mental health staff, retention is crucial to health services. The age profile amongst registered nurses in England is changing, with 40% of the workforce aged 45 or over in 2007 compared to 45.5% in 2017 (Royal College of Nursing, 2018b). As such, creating working conditions that support and retain staff has never been more pertinent. Organisational change initiatives aimed at reducing financial costs need to consider the impact of unforeseen, less quantifiable outcomes. Findings identify a number of unintended consequences of a 12 h shift pattern. Perceptions of reduced quality of patient care, pacing of work and diminished social support arising from a new extended shift pattern, were associated with poorer wellbeing. Moreover, findings revealed how older age, public healthcare commitment and potential for improved work-life balance may moderate the impact of extended shifts on wellbeing.

Extant research has indicated patient-orientated care, whilst demanding, can increase engagement, and foster wellbeing, reducing staff intentions to leave the organisation (Bakker and Sanz-Vergal, 2013; Moloney et al., 2018). If, as identified here, delivery of patient care differs on an extended shift and is transformed in a way that reduces psychological attachment to the job then key issues begin to emerge around employee wellbeing. Wellbeing and burnout amongst healthcare staff has been associated with the quality of patient care (Johnson et al., 2018). Moreover, the direction of the relationship between wellbeing and the quality of patient care may be bidirectional, as positive employee wellbeing may lead to better patient care, whereas an inability to provide satisfactory care may lead to disillusionment and stress amongst staff (Johnson et al., 2018). Additionally, work alienation (powerlessness and meaninglessness) can influence organisational commitment, work effort and quality of life in non-work domains (Tummers and Den Dulk, 2013). As such, imposing an organisational change, which limits opportunities for giving support to patients, and for camaraderie between workers, removes elements of the job that enhance wellbeing and serve to offset negative aspects of a challenging healthcare setting.

Findings suggest a 12 h shift pattern in this context increased the need for stamina, leading to staff pacing their work. Participants struggled to catch up after time away from the workplace, reducing productivity and evoking anxiety as staff felt out of touch with clinical knowledge. This exposes organisational impacts of 12 h shifts in relation to the quality of patient care. Moreover, such pacing behaviours are likely to vary on an individual basis due to innate resilience, and is also of concern in the context of an ageing workforce (Moloney et al., 2018) as physical stamina will become increasingly difficult amongst some older workers (e.g. Phillips and Miltner, 2015; Ryan, Bergin, and Wells, 2017). Additionally, this may lead to increased stress amongst older workers as mental stamina can decrease with age (Valencia and Raingruber, 2010).

Our data reveals how the implementation of a 12 h shift pattern in this context reduced opportunities for social support, which serves to isolate workers. Social support can be a protective factor against strain (e.g.Brough and Pears, 2004), and could be a reason for staying in a job, even if the job is stressful. Support becomes more important in contexts and occupations where emotional support can alleviate the effects of job strain, and in a nursing setting this arguably needs to be embedded in the way wards work. This implies the introduction of a work pattern that disrupts access to support may lead to consequences for employee wellbeing. Conversely, a supportive team climate may lead to positive outcomes of 12 h shifts (Thompson et al., 2017). Data collected between 2009 and 2014 shows a two-thirds increase of agency staff within mental health services (Addicott, Maguire, Honeyman, and Jabbal, 2015). Our findings suggest that working alongside agency workers increased job demands and heightened isolation for core staff. Working with unfamiliar colleagues may lead to negative outcomes of working 12 h shifts (Thompson et al., 2017), despite the integral role agency workers play given ongoing staff shortages.

Given the ageing nursing workforce in the UK (Ryan et al., 2017), our findings raise concerns about the sustainability of a 12 h shift pattern in acute mental health settings, where the environment can be challenging and unpredictable. The inability to sustain physical stamina across a 12 h shift reported by older participants indicate these shift schedules may exacerbate problems with retention of older workers. Moreover, high job demands and lower physical ability has been associated with increased risk of occupational injury amongst the over fifties (Fraade-Blanar et al., 2017). Participants indicated an intention to retire earlier because of 12 h shifts and an unwillingness to return to the relief worker pool if options were limited to 12 h shifts. These findings are consistent with research indicating older nurses value flexible working (Clendon and Walker, 2015). A review of the literature by Uthaman, Chua, and Ang (2016), highlight health and workload as consistent themes relating to early retirement, with shiftwork and flexible schedules cited as both a challenge and an opportunity for the retention of older workers. Thus, enforced changes to shift patterns may be counterproductive in terms of maximising retention of experienced older workers. The loss of more experienced staff has implications in light of an increasing demand for mental health services but also as these staff can help train and mentor newly qualified staff.

As seen in Table 1, many interviewees had worked for the NHS for most of their working lives. This commitment to the sector was evident not only through the number of years worked, but also through their interview responses. Some were struggling with 12 h shifts but, because of their strong commitment to the role, rather than complain, they wanted to be ‘seen to be coping’ to stay in the role, and to support their colleagues. A commitment to the role may shape responses to 12 h shifts and give a false perception of coping and requires some degree of emotional labour, with implications for poorer wellbeing (Brotheridge and Grandey, 2002; Mann and Cowburn, 2005). In addition to the emotional demands the extended working hours may have, the increasing physical demands of the job, coupled with the ageing workforce described above, make this commitment to the profession harder to maintain over time, and could lead to burnout (Brotheridge and Grandey, 2002; Morse et al., 2012).

Data offers insight into why previous evidence signals a preference by employees to work these longer shifts as additional days off compensated staff for working longer shifts. Findings from this study indicate a 12 h shift schedule can create work-life conflicts for some, and personal circumstances will likely lead to differential outcomes relating to work-life balance. Extant research indicates that for work schedules to be beneficial for both parties, employee control is critical (Baillie and Thomas, 2019; Gerdenitsch, Kubicek, and Korunka, 2015; Hyatt and Coslor, 2018; Kossek and Thompson, 2016; Thompson et al., 2017). Conversely, where work-scheduling policies driven by an employer concern for flexibility are imposed, this can create work-life conflicts, at least for some (Hyatt and Coslor, 2018). As such mental health organisations should offer some degree of flexibility and control to local management over the organisation of work in their domain.

### 4.1 Limitations and future directions

The qualitative design of the study prevents the generalisability of findings beyond the sample population. Despite this, a qualitative approach was necessary to explore the meanings behind quantitative outcomes in extant literature. Findings have theoretical generalisability (Ritchie and Lewis, 2006) as the issues raised in this study should have relevance to health services more broadly, particularly in acute settings characterised by high and dynamic job demands. Participants were self-selecting (Saunders, 2012), thus not all employee perceptions were captured. It was not practical for participants to check transcripts for corrections, in part because researchers would need access to participants' contact details or correspondence via ward managers, potentially jeopardising assurances of anonymity. Moreover, being conscious of the high demands of the job, to ask for greater participation might have limited recruitment to the study.

As the introduction of 12 h shifts were compulsory, and commonly perceived as imposed, negative views may have, in part, been a protest response. Having two data collection points, both conducted after any potential ‘honeymoon period’ (Tucker, 2006), addresses this limitation as negative attitudes towards 12 h shifts arising in response to the imposition likely lessen as new working patterns become embedded into practice. In turn, the two data points served to reveal a range of unintended consequences of the change in working hours and the entrenching of these over time. For those who have negative experiences, this presents significant concerns for longer-term wellbeing, and questions the sustainability of these shift patterns in this context given the ageing workforce.

Future research should examine the effect of 12 h shifts over longer periods and evaluate the long run effects of working longer shifts. Mixed methods longitudinal research is essential in order to better understand the longer-term impact on staff and the subsequent implications for service delivery and quality of care. To date there has been little focus on the effect of shift work or a compressed workweek on employee access to and opportunities for social support, something future research could address. In a recent review examining workplace resources relating to employee wellbeing and performance, Nielsen et al. (2017) reported that group level resources, such as supervisor support, have received less attention in the literature compared to organisational and individual level resources. A perceived staff benefit of a 12 h shift system is longer periods away from work. Future research should explore configurations of shift patterns and how this shapes anticipated staff benefits, and examine ‘within’ versus ‘across’ shift stamina in relation to the scheduling of 12 h shifts whilst accounting for moderators and mediators such as age and work-life conflicts.

## 5 Conclusions

Mental health service organisations are suffering workforce shortages and staff working on acute wards are facing increasingly demanding environments. 12 h shift patterns in this context may not be feasible as negative wellbeing outcomes could lead to increased sickness absence, have consequences for retention, or result in staff moving into part-time or non-acute roles. Moreover, long serving staff who intend to work relief shifts following retirement may no longer do so. Of concern is the removal of a longer middle shift altered resources such as social support that may mitigate job demands, and diminishes opportunities to reflect upon professional practice. Findings call into question whether operating a 12 h shift system that rewards staff with additional days off is sustainable in an acute mental health context, as unforeseen consequences and resultant wellbeing outcomes might outweigh reduced wage costs beyond the short term.

## Conflict of Interest

No conflict of interest has been declared by the authors.

## References

[bib0001] Addicott R., Maguire D., Honeyman M., Jabbal J. (2015). Workforce planning in the NHS. http://Www.kingsfund.org.uk/publications/workforce-planning-nhs.

[bib0003] Baillie L., Thomas N. (2019). Changing from 12h to 8h day shifts: a qualitative exploration of effects on organising nursing care and staffing. J. Clin. Nurs..

[bib0004] Ball J.E., Dall'Ora C., Griffiths P. (2015). The 12h shift: friend or foe?. Nurs. Times.

[bib0005] Bakker A., Sanz-Vergal A.I. (2013). Weekly work engagement and flourishing: the role of hindrance and challenge job demands. Journal of Vocational Behaviour.

[bib0006] Bonner G., Lowe T., Rawcliffe D., Wellman N. (2002). Trauma for all: a pilot study of the subjective experience of physical restraint for mental health inpatients and staff in the UK. J Psychiatr Ment Health Nurs.

[bib0007] Brotheridge C.M., Grandey A.A. (2002). Emotional labor and burnout: comparing two perspectives of “People Work”. J Vocat Behav.

[bib0008] CASP (2018). Critical Appraisal Skills Programme (CASP) qualitative checklist. https://casp-uk.net/wp-content/uploads/2018/01/CASP-Qualitative-Checklist-2018.pdf

[bib57] Brough P., Pears J. (2004). Evaluating the influence of the type of social support on job satisfaction and work related psychological well‐being. Int. J. Organ. Behav..

[bib0009] Cassell C. (2015). Conducting research interviews for business and management students.

[bib0010] Clendon J., Walker L. (2015). Nurses aged over 50 and their perceptions of flexible working. J. Nurs. Manag..

[bib0012] Dall'Ora C., Ball J., Recio-Saucedo A., Griffiths P. (2016). Characteristics of shift work and their impact on employee performance and wellbeing: a literature review. Int J. Nurs. Stud..

[bib0013] Dall'Ora C., Ball J., Redfern O., Recio-Saucedo A., Marouotti A., Meredith P., Griffiths P. (2019). Are long nursing shifts on hospital wards associated with sickness absence? A longitudinal retrospective observational study. J. Nurs. Manag..

[bib0014] Dall'Ora C., Griffiths P., Emmanuel T., Rafferty A., Ewings S. (2019). 12-hour shifts in nursing: do they remove unproductive time and information loss or do they reduce education and discussion opportunities for nurses? A cross-sectional study in 12 European countries. J Clin Nurs.

[bib0016] Dwyer T., Jamieson L., Moxham L., Austen D., Smith K. (2007). Evaluation of the 12-hour shift trial in a regional intensive care unit. J. Nurs. Manag..

[bib0017] Edwards D., Burnard P. (2003). A systematic review of stress and stress management interventions for mental health nurses. J. Adv. Nurs..

[bib0019] Fraade-Blanar L.A., Sears J.M., Chan K.C.G., Thompson H.J., Crane P.K., Ebel B.E. (2017). Relating Older Workers’ Injuries to the Mismatch Between Physical Ability and Job Demands. J. Occup. Environ. Med..

[bib0020] Gerdenitsch C., Kubicek B., Korunka C. (2015). Control in flexible working arrangements. J. Pers. Psychol..

[bib0021] Gibbs J., Loretto W., Kowalski T., Platt S. (2011). Case study: organisational change and employee health and wellbeing in the NHS. Work, Health and Wellbeing.

[bib0022] Griffiths P., Dall'Ora C., Simon M., Ball J., Lindqvist R., Rafferty A.-M., Schoonhoven L., Tishelman C., Aiken L.H., RN4CAST Consortium (2014). Nurses’ shift length and overtime working in 12 European countries: the association with perceived quality of care and patient safety. Med. Care.

[bib0023] Hagen J., Knizek B.L., Hjelmeland H. (2017). Mental health nurses’ experiences of caring for suicidal patients in psychiatric wards: an emotional endeavor. Arch. Psychiatr. Nurs..

[bib0024] Harris R., Sims S., Parr J., Davies N. (2015). Impact of 12h shift patterns in nursing: a scoping review. Int. J. Nurs. Stud..

[bib0025] Health Education England (2017). Stepping forward to 2020/21: the mental health workforce plan for England, available at:https://www.hee.nhs.uk/sites/default/files/documents/Stepping%20forward%20to%20202021%20-%20The%20mental%20health%20workforce%20plan%20for%20england.pdf

[bib0026] Hennink M.M., Kaiser B.N., Marconi V.C. (2017). Code Saturation Versus Meaning Saturation. Qual. Health Res..

[bib0027] Hyatt E., Coslor E. (2018). Compressed lives: how “flexible” are employer-imposed compressed work schedules. Pers. Rev..

[bib0028] Johnson J., Hall L.H., Berzins K., Baker J., Melling K., Thompson C. (2018). Mental healthcare staff well-being and burnout: a narrative review of trends, causes, implications, and recommendations for future interventions. Int. J. Ment. Health Nurs..

[bib0029] Kossek E., Thompson R., Allen T.D., Eby L.T. (2016). Workplace flexibility: integrating employer and employee perspectives to close the research-practice implementation gap. The Oxford Handbook of Work and Family.

[bib0030] Knauth P. (2007). Extended Work Periods. Ind. Health.

[bib0031] Mann S., Cowburn J. (2005). Emotional labour and stress within mental health nursing. J. Psychiatr. Ment. Health Nurs..

[bib0032] Mental Health Foundation (2013). *Starting today: Future of mental health services*, available at:https://www.mentalhealth.org.uk/publications/starting-today-future-of-mental-health-services

[bib0033] Moloney W., Boxall P., Parsons M., Cheung G. (2018). Factors predicting Registered Nurses’ intentions to leave their organization and profession: a job demands-resources framework. J. Adv. Nurs..

[bib0034] Morse G., Salyers M.P., Rollins A.L., Monroe-DeVita M., Pfahler C. (2012). Burnout in mental health services: a review of the problem and its remediation. Adm. Policy Ment. Health Ment. Health Serv. Res..

[bib0035] Morse J.M. (2015). Critical Analysis of strategies for determining rigor in qualitative inquiry. Qual. Health Res..

[bib0036] Nielsen K., Miraglia M. (2017). What works for whom in which circumstances? on the need to move beyond the ‘what works?’ question in organizational intervention research. Hum. Relat..

[bib0038] Phillips J.A., Miltner R. (2015). Work hazards for an aging workforce. J. Nurs. Manag..

[bib0039] Rafferty A.E., Jimmieson N.L. (2017). Subjective perceptions of organizational change and employee resistance to change: direct and mediated relationships with employee well-being. Br. J. Manage..

[bib0040] Renwick L., Lavelle M., Brennan G., Stewart D., James K., Richardson M.…., Bowers L. (2016). Physical injury and workplace assault in UK mental health trusts: an analysis of formal reports. Int. J. Ment. Health Nurs..

[bib0041] Richardson A., Turnock C., Harris L., Finley A., Carson S. (2007). A study examining the impact of 12-hour shifts on critical care staff. J. Nurs. Manag..

[bib0042] Ritchie J., Lewis J. (2006). Qualitative Research Practice: A Guide for Social Science Students and Researchers.

[bib0043] Rodriguez Santana I., Anaya Montes M., Chalkley M., Jacobs R., Kowalski T., Suter J. (2020). The impact of extending nurse working hours on staff sickness absence: evidence from a large mental health hospital in England. Int J Nurs Stud.

[bib0044] Royal College of Nursing (2018). Burs Look. Beyond The RNC Bulletin.

[bib0045] Royal College of Nursing (2018). UK Nurs labour Mark. Rev. 2018. https://www.rcn.org.uk/professional-development/publications/pub-007397.

[bib0046] Ryan C., Bergin M., Wells J.S. (2017). Valuable yet Vulnerable: a review of the challenges encountered by older nurses in the workplace. Int. J. Nurs. Stud..

[bib0047] Sainsbury Centre for Mental Health (2005). *Acute Care 2004: A National Survey of Adult Psychiatric Wards in England*, London.

[bib0048] Saunders M., Syman G., Cassell C. (2012). Choosing research participants. Qualitative Organisational Research.

[bib0049] Silverman D. (2016). Qualitative Research.

[bib0050] Thompson L., Schneider J., Duke L.H. (2017). Unregistered health care staff's perceptions of 12 h shifts: an interview study. J. Nurs. Manag..

[bib0051] Tong A., Sainsbury P., Craig J. (2007). Consolidated criteria for reporting qualitative research (COREQ): a 32-item checklist for interviews and focus groups. Int. J. Qual. Healthc..

[bib0052] Tucker, P. (2006). *Compressed working weeks*, International Labour Organization, available at: https://www.ilo.org/wcmsp5/groups/public/—ed_protect/—protrav/—travail/documents/publication/wcms_travail_pub_12.pdf

[bib0053] Tummers L.G., Den Dulk L. (2013). The effects of work alienation on organisational commitment, work effort and work-to-family enrichment. J. Nurs. Manag..

[bib0054] UNISON, (2017). *Struggling to cope – Mental health staff and services under pressure: UNISON's survey report of mental health staff 2017*. https://cdn.basw.co.uk/upload/basw_52349-6.pdf

[bib0055] Uthaman T., Chua T.L., Ang S.Y. (2016). Older nurses: a literature review on challenges, factors in early retirement and workforce retention. Proc. Singap. Healthc..

[bib0056] Valencia D., Raingruber B. (2010). Registered nurses’ views about work and retirement. Clin. Nurs. Res..

